# Mesenchymal (Stem) Stromal Cells Based as New Therapeutic Alternative in Inflammatory Bowel Disease: Basic Mechanisms, Experimental and Clinical Evidence, and Challenges

**DOI:** 10.3390/ijms23168905

**Published:** 2022-08-10

**Authors:** Noemi Eiro, Maria Fraile, Alberto González-Jubete, Luis O. González, Francisco J. Vizoso

**Affiliations:** 1 Research Unit, Fundación Hospital de Jove, Av. de Eduardo Castro, 161, 33290 Gijón, Spain; 2 Department of Anatomical Pathology, Fundación Hospital de Jove, Av. de Eduardo Castro, 161, 33290 Gijón, Spain; 3 Department of Surgery, Fundación Hospital de Jove, Av. de Eduardo Castro, 161, 33290 Gijón, Spain

**Keywords:** Crohn’s disease, ulcerative colitis, cell-free therapy, conditioned medium, extracellular vesicles, exosomes

## Abstract

Inflammatory bowel diseases (IBD) are an example of chronic diseases affecting 40% of the population, which involved tissue damage and an inflammatory process not satisfactorily controlled with current therapies. Data suggest that mesenchymal stem cells (MSC) may be a therapeutic option for these processes, and especially for IBD, due to their multifactorial approaches such as anti-inflammatory, anti-oxidative stress, anti-apoptotic, anti-fibrotic, regenerative, angiogenic, anti-tumor, or anti-microbial. However, MSC therapy is associated with important limitations as safety issues, handling difficulties for therapeutic purposes, and high economic cost. MSC-derived secretome products (conditioned medium or extracellular vesicles) are therefore a therapeutic option in IBD as they exhibit similar effects to their parent cells and avoid the issues of cell therapy. In this review, we proposed further studies to choose the ideal tissue source of MSC to treat IBD, the implementation of new standardized production strategies, quality controls and the integration of other technologies, such as hydrogels, which may improve the therapeutic effects of derived-MSC secretome products in IBD.

## 1. Introduction

Inflammatory bowel disease (IBD) encompasses principally two disorders defined by a chronic inflammation of the gastrointestinal tract: ulcerative colitis (UC) and Crohn’s disease (CD), affecting 1.4 million Americans and 2.4 million Europeans [1]. The incidence and prevalence of IBD, which can lead to life-threatening problems, have increased over the last decades, and is thus a growing public health concern [2,3,4].

**Figure 1:**
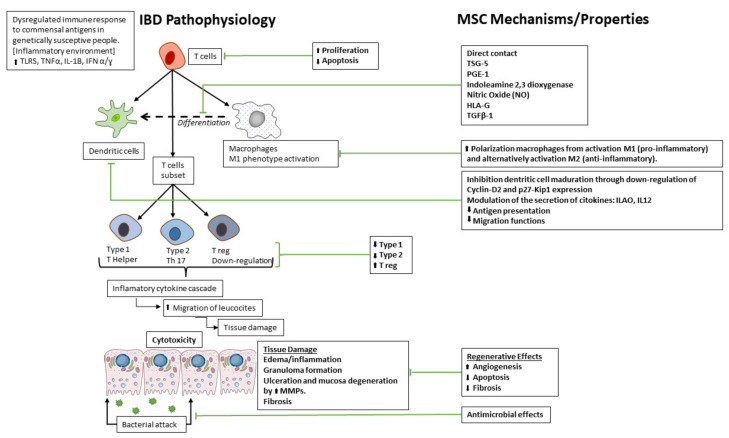
IBD pathophysiology and MSC properties.

IBD has a multifactorial etiology implicating environmental factors, microbiota imbalance, genetic predisposition and mucosa immune defects [5,6]. In the intestine, response to the pathogens produces a controlled inflammation that continues until the pathogen is eradicated; this response is excessive in the IBD [7]. In this scenario, a dysregulate immune response to commensal antigens in genetically susceptible people led to an inflammatory environment characterized by high expression of pro-inflammatory molecular factors such as TLRs, TNF-α, IL-1β and INF-γ, which globally produce both high proliferation and low apoptosis rates of T cells, as well as their differentiation to pro-inflammatory T cells subsets such as dendritic cells, T helper type 1 and M1 macrophages, whereas regulatory T cells (Treg) are down-regulated (Figure 1). The immune imbalance between Treg and Th17 contribute to the progression of colitis [8], since both subset of cellular immune response are interconnected. Th17 express IL-17, a pro-inflammatory interleukin that contributes to tissue inflammation and damage. IL-17 plays a key role in the recruitment and activation of granulocytes and induces the release of pro-inflammatory mediators (IL-1β, TNF-α, IL-6, MMPs, among others) from macrophages leading to exacerbated inflammation [9]. A failure in the regulatory capacity of Treg worsens inflammation. However, Treg inhibit the inflammation through the suppression of effector T cells proliferation and the production of IL-10 and TGF-β [10].

Especially relevant is also the increased polarization of macrophages to the activated pro-inflammatory M1 phenotype which produce high pro-inflammatory cytokines, reactive nitrogen and oxygen species [11]. Taken together, led to an inflammatory cytokine cascade (mainly high levels of TNF-α IL-1β, IL-6, MMPs and nitric oxide) which exacerbate the inflammation [9] by increasing migration of leukocytes and provoking oedema, granuloma formation, ulceration and mucosa degradation, an attack of commensal microbiota resulting in a severe tissue damage and a fibrotic reaction.

Currently, the treatment for IBD starts with 5-ASA agents and antibiotics, depending on the response patients are treated with corticosteroids, immunomodulators and/or biologic agents, but when all this fails, patients require surgery [12]. Such multifactorial factors implicated are obviously difficult to target by using a conventional therapy strategy, which are not completely effective. In addition, all these current therapies are generally noted to have several side effects related to the gastrointestinal tract and liver and severe immune suppressive side effects that impact on lifestyle and predispose individuals to opportunistic infection [13].

For all this, development of more effective treatment for IBD patients, which preserves immune homeostasis and restores the gut barrier, remains to be a challenge [14,15,16,17].

## 2. Stem Cell-Based Therapies in IBD

Cell therapy is defined as the introduction of cells into an organism or tissue in order to treat a disease. In this context, stem cell therapies have significant potential to lead therapeutic development [18]. Stem cells, defined as undifferentiated cells with the capability of differentiate into specific cells of tissues or organs, are characterized as embryonic stem cells, induced pluripotent stem cells (iPSC) or somatic (adult) stem cells, according to their sources. Embryonic stem cells and iPSC are pluripotent cells with the highest multi-differentiation potential; nonetheless, due to ethical concerns and the oncogenic potential, respectively, their clinical use is limited. Somatic stem cells, which are found in many organs and tissues from adults, do not raise special ethical concerns and do not require to be genetically reprogrammed and make them the most used cells for “stem cell-based therapy. Indeed, autologous (from the patient himself) or allogeneic (isolated from a donor) somatic stem cells, such as haematopoietic stem cells (HSC) or mesenchymal (stem) stromal cells (MSC), are used in most clinical trials for IBD.

### 2.1. Haematopoietic Stem Cells

Haematopoietic stem cells transplantation (HSCT) involves the collection and the reinfusion of HSC (from the patient or from a donor) to restore the lymphohematopoietic system [19]. The treatment of non-oncohaematological diseases with HSCT is based on evidences from animal studies and from some cases of remission observed in patients [20].

In the last decade, HSCT has been explore as a new therapy for severe autoimmune diseases, refractory to conventional therapy, including systemic lupus erythematosus and rheumatoid arthritis [21,22]; but also, in severe forms of gastrointestinal pathologies, such as refractory CD. Autologous HSCT to CD patients, with concomitant neoplasms, showed a high efficacy including one case with a remission up to 7 years [23,24,25], but there is the doubt if the maintenance of remission is due to the transplantation (for concomitant neoplasms) in an already quiescent intestinal disease. Later studies report significant remissions or significant symptomatic improvement with these therapies [26,27]. In addition, several aspects were criticized for their designs and unconventional endpoint [28], supporting the need for further controlled clinical trials. In addition, it has been hypothesized that through the plastic properties of stem cells, the self-reactive immune system is eliminated and replaced directly at the source (bone marrow), but the mechanisms of action of auto-HSCT in CD are not well known and understood. Data support that an immunomodulatory “resetting” effect occurs, leading to the restoration of a dynamic balance between self-reactivity and immunological tolerance, probably due to immunologically “naive” stem cells. In fact, after HSCT it was observed a significant decrease of TNF-α and IL-10 secretion and positive TLR-4 cells and an increase of T-regulatory (T-reg) [29].

Allogeneic HSCT seems to reset the immune response and impact on the modification of genetic predisposition to the disease, since HSC with polymorphisms causing the disease are replaced by HSC from a healthy donor [30]. Early reports of allo-HSCT, usually retrospective studies carried out on CD patients with concomitant haematological malignancies, showed the achievement and maintenance of long-term clinical remission (up to 15 years) (Revised by [31]). However, studies focused on IBD patients do not show such a favorable benefit/risk ratio (Revised by [31]). As well, compared with auto-HSCT, allo-HSCT is associated with a higher morbidity and mortality which limits its implementation as a treatment of IBD. In allo-HSCT, despite donor/recipient HLA-matching, both are immunologically different; therefore, after HSCT immune cells from the donor can generate a response against the recipient, called Graft Versus Host Disease (GVHD). This situation requires an adequate immunosuppressive prophylaxis to avoid transplant rejection, which is associated with significant morbidity and mortality [32,33,34]. Nucleotide-binding oligomerization domain 2 (NOD2) mutations or deletion are independent risk factors for CD and GVHD. Host NOD2 expression in the hematopoietic compartment protects against GVHD, as it has been evidenced in bone marrow transplantation in a mouse model [35] and regulates epithelial paracellular permeability [36]. Nevertheless, it has been described that NOD2 contributes to intestinal crypt survival in vivo [37], but when stimulated by muramyl dipeptide, NOD2 in the non-hematopoietic compartment normalized the permeability [36]. So, NOD2 expression in hematopoietic or non-hematopoietic compartment could exhibit a protective role GVHD [38] and regulate intestinal barrier function.

On the other hand, none studies regarding HSCT as a treatment for UC have been published, only some reports when UC patients undergoing HSCT for other indications [31]. Maybe it is due to the identification of UC, but not CD, as an independent risk factor for morbidity and mortality [34].

MSC therapy is considered an interesting therapeutic option for IBD since MSC have low immunogenicity, allowing MSC to escape recognition by the immune system due to low expression of HLA class I and no expression of HLA class II and co-stimulatory molecules (CD40, CD40L, CD80 and CD86) [39,40,41,42].

### 2.2. Mesenchymal (Stem) Stromal Cells

About 50 years ago, MSC were described as a “colony forming unit-fibroblast” from rodent’s bone marrow [43]. MSC can be found in several locations such as bone marrow (BM), adipose tissue (AT), umbilical cord (UC), Wharton’s jelly (WJ), amniotic fluid, placenta, uterus, dermis, skeletal muscle, periosteum, periodontal ligament dental pulp or peripheral blood, among others [44]. In 2006, the “International Society for Cellular Therapy” defined MSC as follows: “(i) MSC must display plastic-adherent capacities; (ii) a simultaneous expression of stromal markers (CD29, CD44, CD73, CD90 and CD105), but the absence of hematopoietic (CD45 and CD14) or endothelial (CD31 and CD34) markers and HLA-DR surface molecules and (iii) an in vitro differentiation potential into osteoblasts, adipocytes and chondroblasts” [45]. MSC are attractive for cell therapies due to their ability to spontaneously migrate to the injured region when the body is damaged, which is named homing effect.

Till the date, over 1000 phase I, II and III clinical trials have been carried out in several pathologies, such as autoimmune and inflammatory diseases [46]. MSC can interact with immune cells and secrete factors, resulting in an immunomodulation. Thus, a change in concept of the therapeutic utility of MSC occurred, from the primarily regenerative effect to the anti-inflammatory and, more recently, to other relevant ones such as angiogenic, anti-oxidative stress, anti-tumoral or anti-microbial [47].

MSC participate and control tissue renewal through their function as sentinels and regulators in tissue homeostasis. Through their ability to regulate oxidative stress, cell proliferation, angiogenesis or apoptosis, MSC influence the tissue microenvironment using paracrine signals, secreting soluble bioactive molecules and/or extracellular vesicles (EVs). The depletion or poor function of MSC has been implicated in several degenerative or autoimmune diseases (such as rheumatoid arthritis [48], systemic lupus erythematosus [49], diabetes mellitus [50] or psoriasis [51]) and in processes associated with aging [52,53]. Consequently, the use of allogenic MSC to restore physiological function has motivated several investigations around the world [18].

#### 2.2.1. MSC and Intestinal Homeostasis

In the intestinal tract, epithelial cells and intestinal mesenchymal cells (iMC) create a physical barrier dividing the microbiome from the underlying tissues and immune system [54]. iMC, a heterogeneous cell population consisting of MSC, myofibroblasts, fibroblasts and pericytes, are present in the subepithelial stroma where they supply a large part of the intestinal structure. More importantly, iMC regulate homeostasis and maintenance of the epithelium barrier [54,55] and foster immunologic tolerance against the commensal bacteria and dietary antigens by the expression of immune-modulating molecules, including programmed death-ligand-1 (PD-L1) [56], chemokines and retinoic acid [57]. As well, recent studies have suggested that iMC play an important role in inhibiting and promoting intestinal inflammation and IBD damage, which can partly, by TGF-β production, contribute to the homeostasis of intestinal epithelium associated with wound healing and mucosal integrity. Furthermore, TGF-β regulates the adhesion molecules and tight junction molecules’ expression and thus adjusts the barrier function of the intestinal epithelium [58]. In this context, iMC isolated from human resected intestine, exhibiting a fibroblast-like morphology and lacking haemopoietic, epithelial and endothelial markers, have showed, in a recent ground-breaking study, to have an immunomodulatory effect and their contribution to epithelial integrity preservation in vitro and in vivo [59]. Remarkably, preconditioning of iMC with TNF and IFN-γ improved their immunosuppressive ability, through indoleamine 2,3-dioxygenase (IDO-1) pathway indicating its role in iMC activity improvement. Moreover, iMC conditioned media enhanced wound healing in vitro. Besides, data show that human iMC isolated from un-inflamed intestine possess tissue-regenerative and immunomodulatory capabilities that could potentially be promoted/restored in order to reduce IBD disease severity. Thus, these data suggest that an impoverishment or dysfunction of iMC and more precisely, MSC may contribute to the pathophysiology of IBD.

#### 2.2.2. MSC and Gut Microbiota

The gastrointestinal tract harbors a complex and dynamic population of microorganisms, known as the gut microbiota, which influence the host during homeostasis and disease, since the microbiota plays a key role in maintaining immune and metabolic homeostasis. The alteration of the gut microbiota, called dysbiosis, is associated with several inflammatory diseases such as IBD [60] and the identification of the functional and integrative map of the microbiota has been proposed to be used as a diagnostic, prognostic and therapeutic tools [61].

Considering that dysbiosis coupled with immune dysregulation could lead to IBD, MSC therapy and microbiota focused therapy like fecal microbiota transplantation (FMT) gain much interest and emerge as a novel approach. Common therapeutic points of both FMT and MSC-therapy in IBD is based on their main functional effects: immunoregulation, tissue damage repair and remodeling, and gut microbiota restoration [62].

It has been evidenced that the crosstalk between MSC and gut microbiota improves the function of each one. Indeed, MSC can promote microbiota normalization in AOM/DSS mice [63], while the microbiota can enhance the immunomodulation capacity of BM-MSC in a DSS mouse model [64]. Hence the need to explore further this approach and its possible mechanisms trough NOD2 [65,66] or EVs [67].

## 3. Therapeutic Potential of MSC in IBD

According to the above hypothesis, many experimental animal studies indicate that the transplantation of MSC has therapeutic potential for IBD. In addition, MSC therapy have also emerged as therapeutic option for refractory IBD patients.

The importance of MSC capabilities for their efficacy on experimental colitis and IBD continues to be studied (Table 1). Several studies shown that MSC ameliorate experimental colitis endpoints, such as body weight changes, bleeding, stool consistency, mortality rate, colon length, colonic inflammation or inflammatory cytokines expression [68,69,70,71,72,73,74,75,76,77,78,79,80,81,82,83,84,85,86,87,88]. MSC from different sources were used in those studies, such as adipose tissue-derived MSC (AT-MSC) [77,78,81,85,89,90], bone marrow-derived MSC (BM-MSC) [68,69,70,74,82,91], umbilical cord blood-derived MSC (UCB-MSC) [71,72,79,92], amnion derived MSC, endometrial derived MSC (E-MSC) [86], tonsil derived MSC, or MSC derived from induced pluripotent stem cells (iPSC) equivalent to AT-MSC [80]. Moreover, MSC were applied by different methods, such as intravenous injection [68,69,70,74,80,82,86,87,93], intraperitoneally administration [69,72,73,75,76,77,79,83,84,85,89,91], mesenteric injection [78] or local injection [69,81,87,88,90,91]. Furthermore, intravenous administration of canine AT-MSC to dogs with IBD induce an improvement of the disease severity and allow to progressively reduce until to suppress steroid dosage [94,95].

A recent meta-analysis, which assessed 46 studies, 28 of which were animal studies (*n* = 567), showed that the disease activity index decreased significantly in MSC treatment groups compared to the control group [96].

**Table 1:** Experimental in vivo studies based on MSC-therapy in IBD.

First Author/Year	Experimental	Cell Surge	Administration Route	Dose of Product	Time	More Relevant Results
Barnhoorn (2020) [97]	DSS-induced colitis	BM-MSC	IP	2 × 10^6^ cells	11 days	MSC after in vivo aggregation show a favorable RNA expression profile for the treatment of colitis. MSC spheroids showed high expression of Ki-67 and low levels of apoptotic marker cleaved caspase-3. Locally applied MSC and MSC spheroids are both able to ameliorate DSS-induced colitis and show similar clinical effects, including improvement in the macro and microscopic IBD score.
Barnhoorn (2020) [97]	DSS-induced colitis	BM-MSC	Endoscopic	2 × 10^6^ cells	4–6 days	Endoscopic injection can be a feasible and effective novel application route for MSC therapy in patients with luminal IBD.
Chao (2016) [71]	TNBS-induced colitis	UC-MSC	IP	10 × 10^6^ cells	14 days	The mortality in UC-MSC-treated TNBS mice was 20% (55% in colitis model); the treatment reduced the inflammation of the transmural area, depletion of epithelial cells and focal loss of crypts. IL-20 and TGF-Beta were significantly higher in UC-MSC-treated mice (*p* = 0.04 and 0.02 respectively).
Cheng (2017) [82]	DSS-induced colitis	BM-MSC/IL-25-BM-MSC	IV	5 × 10^6^ cells	8 days	IL-25-MSC treatment significantly attenuate the colon shortening (12 ± 0.62 cm); IL-25 could enhance immunomodulatory ability of MSC via inhibiting Th17 immune response and promoting the regulation of Tregs cells. However, the study failed to confirm that IL-25 affected the migratory and regenerative capacities of MSC in vivo.
de Aguiar (2018) [84]	DSS-induced colitis	AT-MSC	IP	10 × 10^6^ cells	7 days	ADMSC-treated mice did not present severe reduction in colon length, and presented a reduced tissue damage score index (3); less detachment of mucosa and submucosa layers, low villous blunting and partial preservation of crypt and epithelial integrity. The level of ZO-1 expression in the colon was re-established in ADMSC-treated mice. Significant reduction of IFN-gamma and TNF-Alpha, and reduction of IL-6 and MCP-1 protein levels. ADMSC treatment reduced DCs and macrophages presence in the colon.
de Aguiar (2018) [84]	DSS-induced colitis	AT-MSC	IP	2 × 10^6^ cells	7 days	DMSC ameliorated the severity of DSS-induced colitis, reducing colitis pathological score and preventing colon shortening.
de la Portilla (2013) [98]	TNBS-induced colitis	AT-MSC	Local	60 × 10^6^ cells	24 weeks	First study which shows the homing migration of MSC to areas of experimentally-induced colitis following rectal installation.
de la Portilla (2018) [90]	TNBS-induced colitis	AT-MSC	Local	2 × 10^6^ cells	10 days	There were no differences in component rectal wall thicknesses with a higher Hunter score in the treated group compared with the controls.
Fu (2017) [78]	TNBS-induced colitis	AT-MSC	Mesenteric injection	2 × 10^6^ cells	6 days	Dcreased the weight loss and DAI score, MPO activity; moreover relieved colitis, decreased colonic shortening, inflammatory cell infiltration and mucosal ulceration. Reduced levels of ROR(lamda)t and IL-17A; inhibited STAT3 phosphorylation, but increased STAT5 phosphorylation.
González-Rey (2009) [77]	DSS-induced chronic colitis	AT-MSC	IP	10 × 10^6^ cells	27 days	AT-MSC treatment protects against DSS-induced acute colitis as well as chronic severe colitis (*p* 0.01 and *p* 0.001 respectively); reduces colonic inflammatory responses in DSS-induced chronic colitis (*p* 0.001).
Gregoire (2018) [99]	Fistulising Crohn’s disease	AT-MSC	Single intrafistular injection	3 × 10^6^−30 × 10^6^ cells	8 weeks	6/8 fistulas healed, 2/8 improved.
Heidari (2021) [100]	DSS-induced colitis	AT-MSC/MSC-CM	IP	10 × 10^6^ cells	34 days	There was no significant difference in the survival rate among the study groups; however, there was a significant increase in terms of the colon length (*p* 0.005). In the treated mice the level of mucosal damage was significantly lower (*p* 0.005), and the structure of the crypts also showed improvement of tissue healing.
Heidari (2021) [100]	DSS-induced colitis	AT-MSC	IP	2 × 10^6^ cells	34 day	The regulatory effects of AT-MSC and their CM in inflammatory conditions because of colitis
In Kap (2010) [93]	DSS-induced colitis	MSC	IV	1 × 10^6^ cells	7 days	Anti-addressin Ab coating on MSC increased cell delivery to inflamed colon and increased the efficacy of MSC treatment of IBD.
Jianxia Hu (2016) [101]	Luminal Crohn’s disease	UCB-MSC	IV	0.5 × 10^6^ cells	3 month	30/36 patients showed good response and diffuse and deep ulcer formation and severe inflammatory mucosa were improved markedly.
Lee (2016) [70]	DSS-induced colitis	BM-MSC	IV	10 × 10^6^ cells	33 days	IL-10 production was upregulated by about 10-fold in BM-MSC-treated mice and showed a preventive effect on weight loss.
Lee (2016) [70]	DSS-induced colitis	BM-MSC	IV	30 × 10^6^ cells	33 days	Infusion of BM-MSC at the onset of disease exerted preventive and rapid recovery effects.
Lee (2018) [79]	DSS-induced colitis	UCB-MSC	IP	2 × 10^6^ cells	12 days	The survival rate was further increased by co-treatment compared to UCB-MSC or MIS416 single treatments; colon lengths were significantly increased in co-treatment; colonic inflammation was more effectively resolved by co-treatment with MSI416 and UCB-MSC, and only co-treatment markedly decreased fibrosis and enhanced tissue regeneration. Exposure to MIS416 increases the number of immune cells via activation of CD14+ macrophages.
Legaki (2016) [83]	DSS-induced colitis	Amniotic fluid-MSC	IP	1.5 × 10^6^ cells, 200 μL/dose	7 days	CM treatment significantly decreased the extension and severity of the inflammation in comparison to the DSS-treated mice; the relative expression levels of IL-10 mRNA were significantly increased, similarly TNF-a and IL-1B levels were decreased at mRNA level. Additionally, TGFb1 was significantly higher (*p* 0.0001).
Mao (2017) [102]	DSS-induced colitis	UCB-MSC	IV	400 μg exosomes/1.3 × 10^6^ cells	11 days	Exosomes from MSC have profound effects on alleviating DSS-induced IBD and may exert their impact through the modulation of IL-7 expression in macrophages.
Martín (2018) [103]	TNBS-induced colitis	AT-MSC	Local	10 × 10^6^ cells	11 days	Submucosal injection of human ASCs ameliorates the course of TNBS colitis in immunocompetent rats.
Martin Arranz (2018) [81]	TNBS-induced colitis	AT-MSC	Endoscopic	10 × 10^6^ cells	11 days	The endoscopic score improved in the ASC group by 47.1% ± 5.3% vs. 21.8% ± 6.6% in the vehicle group.
Miyamoto (2017) [87]	TNBS-induced colitis	AT-MSC	IV and Local	1 × 10^6^ cells IV and 400 μL Local	7 days	hAMSC transplantation significantly decreased the number of neutrophils, attenuated acute inflammation, suppressed the expression levels of inflammatory mediators in the colons; in the TNBS-CM gel group ulcers were shallow and bleeding was not detected, therefore improved endoscopic score. In the gel group mRNA expression levels of TNF-Alpha, CXCL1, CCL2 and IL-6 were increased.
Molendijk (2015) [104]	Fistulising Crohn’s disease	BM-MSC	Single intrafistular injection	10, 30, 90 × 10^6^ cells	6, 12, 24 weeks	At week twelve, 3 of 9 individual fistulas had healed in group 1 (33.3%), 6 of 7 had healed in group 2 (85.7%), 2 of 7 had healed in group 3 (28.6%), and 3 of 9 had healed in the placebo group (33.3%).
Pak (2018) [88]	DSS-induced colitis	BM-MSC/AT-MSC	Endoscopic	8 × 105 cells/1.1 × 10^6^ cells	1–3 days	The success rate was 37.60% for AT-MSC group and 35.20% for BM-MSC group.
Panés (2016) [105]	Fistulising Crohn’s disease	AT-MSC	Single intrafistular injection	120 × 10^6^ cells	24 weeks	Remission in the ITT (53 of 107 [50%] vs. 36 of 105 [34%]; difference 15.2%, 97.5% CI 0.2–30.3; *p* = 0·024) C × 601 vs. placebo.
Panés (2018) [106]	Fistulising Crohn’s disease	AT-MSC	Single intrafistular injection	120 × 10^6^ cells	52 weeks	C × 601 achieved combined remission (56.3%) vs. controls (38.6%) (a difference of 17.7 percentage points; 95% CI 4.2–31.2; *p* = 0.010).
Park (2018) [107]	DSS-induced chronic colitis	AT-MSC	IP	10 × 10^6^ cells	20 days	In DSS-induced chronic colitis model, hASCs decreased the frequency of macrophage transition, specially M1 macrophages. The results suggest that PGE2, produced by co-culture of ASCs and THP-1, reduces M1 population.
Park (2018) [107]	DSS-induced colitis	AT-MSC	IP	2 × 10^6^ cells	20 day	ASCs can suppress the inflammatory response by controlling the macrophage population, and ASCs may be therapeutically useful for the treatment of IBD.
Pouya (2018) [75]	DSS-induced colitis	MSC-CM	IP	500 μL, ×3	10 days	After infusion, colon inflammation was reduced and histopathological analysis showed a decrease in mucosal degeneration.
Song (2017) [72]	DSS-induced acute/chronic colitis	MSC-Ex/UC-MSC	IP	10 × 10^6^ cells; 150 μg/mouse	36 days	MSC-Ex ameliorates the clinical parameters in DSS-induced colitis; the treated group showed significantly less MPO activity. The level of IL-17 was significantly decreased, whereas those of IL-10 and TGF-Beta1 were increased. MSC-Ex is superior to UC-MSC in chronic IBD models, without differences in the colon length.
Song (2018) [85]	DSS-induced colitis	canine AT-MSC	IP	2 × 10^6^ + TSG-6 siRNA	10 days	AT-MSC-secreted TSG-6 reduced inflammatory response and apoptosis in the colon; intraperitoneally infused cAT-MSC did not migrate to the inflamed colon; increased M2 macrophages in the inflamed colon.
Soontararak (2018) [80]	DSS-induced colitis	iMSC/AT-MSC	IV	3 × 10^6^ cells	19 days	The clinical illness scores were significantly reduced (iMSC-treated *p* = 0.003, adMSC-treated *p* = 0.001); colonic tissues from mice treated with either iMSC or adMSC exhibited an overall reduction in transmural inflammation, with significantly less infiltration of inlammatory cells in the lamina propria, diminished mucosal ulceration and decreased mucosal collapse and granulation tissue formation.
Tanaka (2008) [68]	DSS-induced colitis	BM-MSC	IV	5 × 10^6^ cells	7 days	In the rectum of treated rats the mRNA expression of TNF-alpha and IL-1Beta was markedly decreased to (43.7 ± 25.5% *p* 0.05 and 14.5 ± 12% *p* 0.01 respectively), as well as COX-2 16.5 ± 15.2% (*p* 0.01).
Tanaka (2008) [68]	DSS-induced colitis	MSC	IV	5 × 10^6^ cells	7 days	Exogenous MSC accumulated in inflamed tissues and ameliorated DSS-induced colitis via a local anti-inflammatory action.
Wang (2016) [108]	DSS-induced colitis	BM-MSC	IP	0.5 × 10^6^ cells	10 days	Intraperitoneal injection is the best delivery way for MSC: showed better mucosa recovery and higher cell engraftment at inflamed colon.
Wu (2018) [109]	DSS-induced colitis	UCB-MSC	IV	400 μg UC-MSC	11 days	Exosomes from hucMSC have profound effects on alleviating DSS-induced IBD and may exert their function by regulating the ubiquitin modification level.
Xu (2018) [86]	DSS-induced colitis	Endometrial regenerative cells (ERC)	IV	3 × 10^6^	10 days	ERC treatment significantly reduced the levels of TNF-Alpha, IL-1Beta and IL-6; ERCs downregulated the expanded Th1 and Th17 cells in colitis, and elevated the proportion of Tregs in lymphocytes; ERCs inhibited B-cell activation, differentiation and IgG production in colitis. ERC treatment enhanced the concentration of IL-10 in the colon and spleen, as well as CD1dhiCD5 + B cells in the spleen, peritoneal cavity and MLN.
Yu (2017) [73]	DSS-induced colitis	Tonsil-MSC	IP	20 × 10^6^/40 × 10^6^ cells	30 days	Co-culture with T-MSC clearly inhibited the PMA-stimulated proliferation of splenocytes by 60%; TMSC [×4] treated mice’s survival rate was improved to that of the normal. TMSC [×2] injection also significantly improved the survival rate to 89% of the control. TMSC [×4] treatment inhibits DSS-induced colon shortening; TMSC injection does no inhibit histopathological alterations in the distal colon in the chronic colitis mouse model, although it ameliorates IL-1Beta and IL-6 mRNA production in chronic colitis mice.

IP: Intraperitoneal; IV: intravenous.

### 3.1. Potential Therapeutic Mechanisms of MSC in IBD

#### 3.1.1. Anti-Inflammatory Effects

MSC modulate immune adaptive cells from a high amount of T-cell effector to a regulatory T (Treg)-rich microenvironment, through paracrine factors like transforming growth factor beta (TGF-β) [110], hepatocyte growth factor (HGF) [111], prostaglandin E2 (PGE2) [112], nitric oxide (NO) [113], and indoleamine 2,3-dioxygenase (IDO) [114]. IL-10 is another MSC-generated immunoregulatory cytokine which contributes to bowel homeostasis, its deficiency aggravates DSS-mediated colitis whereas its supplementation could become an alternative treatment for IBD [115]. As a result of these soluble factors, MSC exert an anti-inflammatory effect on different types of immune cells. In this sense, it has been reported that MSC downregulate Th1 and Th17 responses and upregulate Th2 and Treg-mediated responses, which contribute to the improvement of colonic inflammation [116,117]. Also, MSC express NOD2, and its binding to muramyl dipeptide (MDP) ligand, enhances the secretion of anti-inflammatory factors such as PEG2 and IL-10, and stimulates the production of Tregs. In this regard, the anti-inflammatory effect of UC-MSC was enhanced by the activation of NOD2 through the activation of COX-2 signalling, which reduced disease severity in a mouse model of colitis [118]. Furthermore, co-administration of MIS416 (a microparticle that activates NOD2 and TLR9 signalling) and UC-MSCs improved therapeutic efficacy of MSC [79]. Additionally, there are other specific mechanisms associated with anti-inflammatory effects through EVs derived from MSC, as discussed below.

#### 3.1.2. Regenerative Effects

MSC secrete several biological molecules such as cytokines, growth factors and lipids, which impact on tissue renewal [107]. Thus, paracrine signalling of MSC was proposed as the primary mechanism for their regenerative effect on parenchymal and mesenchymal cells [119], acting on processes implicated in tissue regeneration, like migration, immunomodulation and re-epithelization [47]. A correct tissue regeneration requires new blood vessels formation for the administration of oxygen, nutrients, and growth factor; in this sense, it was suggested that the induction of angiogenesis is another one of the principal mechanisms of action of MSC on tissue regeneration. Through the secretion of molecular factors, such as VEGF, PDGF, ANG-1 y 2, EGF, FGF, TGF-β1, TGF-α, MCP-1, CXCL5, and MMPs [44,120], MSC increase the proliferation and migration of endothelial cells. It has been described that MSC through growth factors, cytokines, and EVs can promote the survival and regeneration of colonic epithelial cells [121] and limit colonic tissue damage such as atrophy, inflammation, and dysplasia [122].

#### 3.1.3. Antifibrotic Effects

A pro-fibrotic state, induced by oxidative stress, inflammation, and aging, is related to numerous progressive and terminal illnesses. This architecture entails the over-deposition of extracellular matrix (ECM) proteins, such as fibronectin and collagens I and III, resulting in altered biological functions and reduced tissues regeneration capacity [47,123]. In vivo studies disclose an anti-fibrotic effect of MSC like against skeletal muscular fibrosis, primarily through MMP-1 [124], or kidney or liver fibrosis via VEGF and HGF secretion, and further eliminates TGF-β1-induced fibrotic changes [125,126], with TGF-β1/Smad route which is a major pathogenic mechanism in tissue fibrosis [127,128]. MSC secretome and EVs have also an antifibrotic effect by containing growth factors and cytokines, such as HGF, TGF-β3, TNF-α, and IL-10 [129,130,131], and through the inhibition of dermal fibroblast–myofibroblast transition by blocking the TGF-β1/Smad2/3 signalling pathway, respectively [129,132,133].

Recently, in a phase I-II pilot trial it was assessed safety and efficacy of local MSC injection to treat CD strictures reporting that it was well tolerated and may offer a benefit. This is an interesting discovery since intestinal fibrosis is a common complication in IBD, especially in CD, causing bowel constriction and obstruction.

#### 3.1.4. Anti-Apoptotic Effects

The study of anti-apoptotic mechanisms of MSC in various organ injury models is of great interest [47]. AT-MSC induce the upregulation of the cell proliferation marker Ki67 and the antiapoptotic markers BCL2 and SURVIVIN, and the downregulation of apoptosis indicators like TUNEL, annexin V, CASPASE3, and CASPASE9 [134]. Also, exosomes from BM-MSC containing miRNA-146a-5p reduce neuron apoptosis and inflammation through the downregulation of IRAK1 and NFAT5 expression [135]. For IBD, it has been proposed the use of MSC expressing IL-37b, cells transduced with an adenovirus vector expressing IL-37b, since they can inhibit the inflammatory process and cellular apoptosis [108]. Also, it has been evidenced that EVs from BM-MSC suppress the apoptosis through the reduction of the cleavage of caspase-3, caspase-8 and caspase-9 in an animal model [136].

#### 3.1.5. Anti-Oxidative Stress Effects

Oxidative stress is a common and crucial pathophysiological mechanism of several illnesses [137]. Oxidative stress is due to the imbalance of pro-oxidants, or free radicals, and antioxidants, inducing a structural modification of lipids, proteins, and DNA causing pathology or damage to a cell or tissue [138]. Reactive oxygen species (ROS), such as superoxide anion (O_2_ (−)), hydroxyl radical ((·)OH), and hydrogen peroxide (H_2_O_2_) are the most studied free radicals [139,140]. The dynamic balance of ROS production and metabolism is crucial for tissue homeostasis, when the disruption of this balance lead to oxidative stress and tissue damage [141]. Although the relationship between the immune system and oxidative stress is not elucidated, leukocytes and pro-inflammatory mediators increase free radicals composition and disrupt the redox environment inducing a positive feedback loop [142]. Neutrophils are contributors to oxidative stress in inflammation, since they have an extensive activity of myeloperoxidase (MPO), which play a significant role in oxidative stress by catalyzing H_2_O_2_ to hypochlorite [143,144].

Several studies reported the resistance of MSC to oxidative injury, related to the constitutive expression of antioxidant enzymes (SOD1, SOD2, catalase (CAT), glutathione peroxidase (Gpx), high levels of the antioxidant glutathione (GSH)) [145], and other proteins such as HSP70 and SIRT, which may also play a role in the resistance to oxidative stress [146]. Antioxidant effects of MSC have been observed in vitro and in vivo using multiple disease models (aging, gastrointestinal inflammation, ischemic injuries) [47] identifying various mechanisms such as retrieval of free radicals, promotion of native antioxidant defenses, immunomodulation via ROS suppression and endowing functional mitochondria to damaged cells [137]. Also, MSC can decrease ROS and MPO activity in monocytes and macrophages, reducing their pro-inflammatory phenotype [147,148]. In a rat model of colitis, EVs from BM-MSC reduce oxidative perturbations increasing antioxidant enzymes (SOD and GSH) and decreasing in the activity of malondialdehyde (MDA) and MPO [136]. All these data indicate that to prevent oxidative injury, MSC induce an immunomodulation depending on their antioxidant properties.

#### 3.1.6. Antimicrobial Effects

MSC resistance to viral infection is mainly due to the expression of interferon (IFN) and IFN-stimulated genes (ISGs) [149] which hamper infection before viruses can cross the cell membrane, as demonstrated to be so for influenza A virus and SARS coronavirus [150]. MSC can act directly by releasing antimicrobial peptides [151,152] leading to cell destruction, interfering with membrane integrity, inhibiting DNA, RNA or protein synthesis; and engaging with specific intracellular targets [153,154,155,156]. LL-37 is one of this kind of peptide showing a broad spectrum of antibacterial endeavors against both Gram-negative and Gram-positive bacteria [157,158,159] and antifungal [160] and antiviral activities [161]. Also, LL-37 is related to the modulation of Toll-like receptors (TLRs), which is a precursor of the immunomodulatory activity of MSC [162]. Also, MSC can act by releasing soluble proteins with a known antimicrobial effect such as interleukin-10 (IL-10), prostaglandin E2 (PGE2), tumor necrosis and factor-alpha (TNF-α) [163], IDO [164], and interleukin-17 [165]. In addition, the secretoma of MSC (conditioned medium) has an anti-microbial activity, including against *E. coli* and *S. aureus* [152,166], *E. epidemidis* [166], *Vibrio cholerae* [129,167], *P*. *aeruginosa* [168], *Mycobacterium tuberculosis* [169], *Acinetobacter baumannii* [170], and several *Candida* species [171,], including effects against biofilm build-up [172,173].

#### 3.1.7. Anti-Tumor Effects

Cell communication between tumor cells and stromal environment is key to cancer pathophysiology. Pro- or anti-tumorigenic effect of MSC depends on the tissue origin of MSC and the kind of tumor [174,175] and MSC from uterus and pregnancy-related tissues seems to have a wider antitumor effect, which make them good candidates for MSC-based therapy for cancer [176].

MSC produce high level of cytokines, such as IFN-α, DKK-1/3, IL12, TRAIL, TNFSF14 (also known as LIGHT), FLT-3 ligand, CXCL10 and LAP, known to inhibit tumor growth in vivo in breast cancer cells [177,178,179,180]. Also, the antitumoral effect of MSC may be, in part, due to the presence in the secretome of tissue inhibitors of matrix metalloproteinase (TIMP-1 and TIMP-2) [177,181], associated with the migration suppression and invasion of cancer cells.

Other mechanism used by MSC to take effect on cancer cells is through EVs, since cancer cells internalize EVs more efficiently than normal cells [182,183]. It has been reported that EVs from human UC-MSC inhibit the development of bladder carcinoma, reducing the phosphorylation of Akt protein kinase and increasing cleaved caspase-3 [184], and EVs from AT-MSC suppress ovarian cancer cells proliferation through their exosomal miRNA content [185].

#### 3.1.8. IBD and Colon Cancer

It was noted that anti-inflammatory drugs can effectively reduce the risk of developing colitis associated with colorectal cancer (CAC) [186]. However, long-term use of these therapies is restricted due to their potentially fatal adverse reactions [187]. Therefore, there is a pressing need to explore novel anti-inflammatory therapeutic approaches to cancer.

Several studies showed that MSC inhibited CRC initiation in an azoxymethane (AOM)/dextran sulfate sodium (DSS) mouse model [188,189,190], which closely mimics the mechanisms of human CAC [191]. Furthermore, several mechanisms for this effect have been identified, such as through IL-6-STAT3 signalling [188] or regulating the differentiation of Treg cells by Smad2 [190].

Recently, MSC have been shown to migrate to the intestine following intraperitoneal injection and prevent the appearance of colitis-associated colorectal cancer. This inhibition effect was characterized by a decrease in weight loss, a longer colon length, and a reduction in the number of tumors. Moreover, MSC also reduced tumor cell proliferation and caused tumor cell apoptosis [63]. This study also found that MSC did not change the abundance, but rather the diversity and composition of the intestinal microbiome. The analysis in genus levels revealed that an injection of MSC increased the abundance of potentially beneficial bacteria and reduced the amount of potentially harmful bacteria in the gut microbiome of mice. It is worth noting that the wealth of *Parabacteroides*, *Staphylococcus*, *Acetatifactor*, *Intestinimonas*, and *Candidatus Saccharimonas* was increased after administering MSC. *Parabacteroides* in faecal matter was displayed to bind inversely to colon tumor numbers and has anti-inflammatory and anticancer properties [192]. *Staphylococcus* is a commensal strain allegedly apoptosis trigger [193] and may protect from neoplasia [194]. Other research also demonstrated that MSC attenuate gut microbiota dysbiosis [80]. These are interesting discoveries as intestinal microbes are implicated in the intestinal defense function and immune system maturation, whereas gut microbiome dysbiosis plays a role in the pathogenesis of IBD and CRC [195]. Consequently, all of these data suggest that MSC may be a promising strategy for colitis-associated colorectal cancer.

### 3.2. Clinical Trials

#### 3.2.1. Clinical Trials on MSC Transplantation in IBD

Over 1000 clinical trials based on MSC therapy are registered on the NIH Clinical Trial Database (https://ClinicalTrials.gov/), of them 491 trials (47.1%) for immune disorders and of these last, 34 trials conducted on IBD patients [196].

Trials on IBD used autologous or allogeneic (most of them) MSC transplantation by local injection to treat fistulizing CD or by systemic intravenous infusions to treat UC or luminal Crohn’s disease. Allogeneic MSC are more convenient since they can be expanded in mass to obtain fully characterized and enough cells dose prior to administration.

#### 3.2.2. MSC in Fistulizing and Perianal CD

Fistula relapse occurs in approximately 30% of CD patients and is associated with abscess formation, decreased organ function and reduced quality of life [197]. Although the pathogenesis of complex perianal fistula is unknown, it is assumed that it is due to an immune-mediated disturb in the context of CD. The perianal fistula treatment aims to block fistula from draining, to avoid complications such as the fecal incontinence, and reach a durable closure. Currently, the treatment includes conventional drugs (antibiotics and immunomodulators), biological therapy based on anti-TNF antibodies (e.g., infliximab, adalimumab, and certolizumab) and surgical treatments [198], but they fail to maintain a durable closure of the fistula.

In 2003, the first study on MSC-therapy for perianal fistula on CD patients was published reporting a successful healing after local administration [199]. Since then, more phase I/II studies were carried with AT-MSC and BM-MSC, so autologous [200,201,202,203,204] as allogeneic [97,98,104,205], transplanted after ex vivo expansion. In these studies, peri-fistula injections of MSC promoted healing in many cases of complex perianal fistulas in CD patients, refractory to conventional or biological therapy.

In 2016, Panés et al. completed the ADMIRE trial, a phase III randomized, double-blind, placebo-controlled study using the above mentioned Cx601 product (now called darvadstrocel) for allogeneic AT-MSC transplantation in CD patients with complex perianal fistulas [105]. This randomized clinical trial involved 212 CD patients, conducted in 49 hospitals. A higher number of patients treated with Cx601 vs. placebo achieved remission at 24 weeks. The benefit over placebo was sustained 52 weeks after local injection [106]. Nevertheless, one randomized clinical trial reported no differences between treated and control patients after one year of follow-up [206].

Recently, a meta-analysis on CD perianal fistulas has reported that MSC therapy increased healing rate compared to control groups (OR 0.379, 95% CI 0.152–0.947), existing significant differences between autologous and allogenic MSC (69.4% and 50.7% (*p* = 0.020), respectively), but CD perianal fistulas treated with MSC achieve an overall healing rate of 64.1% [207]. In addition, one more recent meta-analysis evidenced the effective and safety application of MSC combined with fibrin glue for complex perianal fistula (CD or non-CD) evidencing an angiogenic effect of fibrin glue which together with MSC differentiation capacity show a synergistic effect on fistula’s healing [208]. Nevertheless, studies evaluated in this meta-analysis seems to be heterogeneous regarding fistula closure definition and MSC administration and doses. There is a controversial regarding these results which will be referred below in the section of “limitations of MSC therapy in IBD”.

#### 3.2.3. MSC Transplantation in Luminal Crohn’s Disease

Until today, only BM-MSC and MSC from neonatal tissues have been employed as systemic therapy to treat luminal CD. Two phase I trials have evaluated the intravenous administration of autologous BM-MSC in luminal CD patients, but the results are disappointing, since the therapy failed to achieve clinical remission [209,210]. Similarly, phase I-II trials on intravenous administration of allogeneic BM-MSC in moderate-severe refractory CD only showed a clinical remission rate of 32% [99,211,212,213]. Regarding the use of MSC from neonatal tissues such as placenta, umbilical cord, or amnion in clinical trial, the results are preliminary or pending to be published [214,215,216].

A recent meta-analysis, which includes 18 human trials on CD patients (*n* = 360), reported that MSC therapy induces maintained remission for 3 to 24 months and decreases activity index, endoscopic index of severity and simplified endoscopy score. But also concluded the need to carry out high-quality randomized controlled clinical trials and basic research [96].

#### 3.2.4. MSC Transplantation in Ulcerative Colitis

Although several clinical trials were conducted with MSC therapy in more than 200 UC patients, few of them are fully published and the clinical remission rate is doubtful due to imprecise definitions [101,217,218,219]. Recently, a meta-analysis reviewed 7 trials (n = 216 MSC treated patients) of whom 2 studies (n = 33 patients) use submucosal MSC administration by colonoscopy, 3 studies (n = 136 patients) opted to intravenous infusion and for the remaining 2 (n = 47) the administration route is not available [121]. Four of these studies do not include a control group and only one was a randomized controlled trial. Although the healing rate, the main outcome of trials, was not clearly defined in 4 single-arm study the overall healing rate was 0.787 and in the other 3 studies the healing rate was 0.791 and 0.853, respectively for the 2 group of treatment (MSC vs. 5-ASA and MSC + 5-ASA vs. placebo + 5-ASA).

## 4. Limitations of MSC Therapy in IBD

The limitations associated with MSC clinical use are based on the difficulty to provide a consistent supply complying with the required cell stability, the high cost related to the isolation and handling process and adverse events such as the infusional toxicity [220] or cellular rejection [221,222]. Despite of some positive outcomes [101], until now disappointing results have been found with regards to the systemic administration of MSC to patients with IBD [223]. Safety of systemic application of MSC, still need to be explored since several clinical trials reported aggravation of UC or CD [224]. Nevertheless, the optimal source of MSC, route of administration and dosage have to be resolved yet. [223,224]. In addition, the basic limitation of this therapy is the great number of cells required for transplantation [225,226,227,228,229].

Instead, regarding CD treatment, there are still many unsolved questions that are concerning: (i) differences in the dose of MSC injected and the number of injections; (ii) the most appropriate technique for the MSC transplant is still controversial in the literature (e.g., direct injection, injection with fibrin glue, and delivery on a fistula plug); (iii) the time point of fistula healing; (iv) criteria for defining fistula healing; (v) the best tissue to obtain the MSC; (vi) unclear treatment mechanisms; and (vii) eligible patients were all at least 18 years of age. In the future, an optimized and standardized treatment scheme should be adopted to enable patients to achieve long-term healing [208].

### Regulatory Aspects

Over 1000 clinical in more than 40 countries have been conducted with MSC or their derivatives but only nine MSC-based products have been approved [230]. Of them, two are treatment for IBD patients such as allogenic AT-MSC to treat complex perianal fistulas in adult CD patients, approved by the European Medicines Agency of the European Union, and autologous AT-MSC for CD patients approved by the Korean Ministry of Food and Drug Safety.

Despite the clinical efficacy demonstrated in various clinical trials, few MSC products have been approved as a treatment, indicating on one side, significant differences in regulations between countries [231] and on the other side, obstacles to establish a transition from preclinical platforms to platforms clinics [232].

There is an important lack of agreement on relevant characterization criteria for clinical application since the current criteria were established in 2006 and did not consider some properties as immunomodulation which was demonstrated subsequently. Moreover, carry out a double-blind randomized clinical trial, considered the gold standard, is much more challenging with MSC-based products than with a classical drug, specially to dose, timing and delivery route testing [233]. Some changes are taking place to define clinical effectiveness for future clinical trial and drug approval [230].

## 5. New Perspectives for MSC Therapy in IBD

### 5.1. Importance of the Type of MSC

On the other hand, there are recognized limitations of MSC-based therapies related to the transplantation of proliferating living cells including immunological incompatibility, tumorigenicity, transmissible infections, and the initiation of the process of MSC senescence [18,234,235]. MSC administration can also induce thromboembolism [236], since they express procoagulant activity (PCA) that can initiate the coagulation process, when in contact with blood [237]. However, despite the mentioned limitations, MSC administration was safe [238,239].

The functional alterations evidenced in MSC from patients affected by systemic diseases such as diabetes mellitus, obesity, systemic lupus erythematosus, and rheumatoid arthritis, make them non-ideal donors. Also, the aging of MSC is a limiting factor as older cells lose stem cells competence and may enter in senescence. MSC aging can induce a senescence-associated phenotype and produce proinflammatory cytokines, which inhibits the regenerative process [240]. In this sense, the culture of MSCs induces a progressive loss of self-renewal and multipotency capacity [241,242], allowing no more than 30 to 40 population doublings, which constitutes a limitation for research and cell therapy [46,243,244,245]. This last limitation can be solved with the immortalization process [246] through telomere maintenance and suppression of p53- and Rb-mediated pathways, which allows the preservation of MSC phenotype and multipotency and increase the proliferation rate. In addition, both MSC morphology and functionality were similar after transduction of immortalization genes [245,247].

MSC functionality differs depending on their tissue origin, as it was reported between AT-MSC and BM-MSC regarding the proliferation and differentiation capacity, or paracrine mechanisms, like the secretion of pro-angiogenic factors [176]. With regard to anti-inflammatory effects, heterogeneity of MSC from different sources often secrete varying levels of soluble factors and thus exert diverse suppressive effects [229]. As it was described above, different tissue sources of MSC were employed depending on the clinical application. In this sense, AT-MSC were predominantly used and exhibited successful healing in fistulizing and perianal CD whereas BM-MSC seem induce clinical remission in UC patients. Some sources of MSC may be attractive for specific indications, such as MSC from reproductive tissues by their anti-tumor activity [180,248], or human uterine cervical stem cells (hUCESC) for their immunomodulatory [180] and antifungal activity [171], or MSC from dental pulp by neurological disorders [249].

The genetic manipulation of MSC provide a possibility to improve some of their capabilities; as it has been demonstrated with the incorporation of anti-inflammatory genes to MSC (such as IL-10; IDO; or Foxp3) or the induced overexpression of factors leading to cell survival; angiogenic; neuroprotective; or anti-cancer activities [176]. Nevertheless; biological attributes of the cell and gene therapy products; such as MSC integrating viral vectors; usually replication-deficient such as adenovirus; adeno- retrovirus or lentivirus; are associated with toxicity and carcinogenicity/tumorigenicity potential [250] which limit clinical application. It has been reported that CRISPR-Cas system may improve MSC effect [251,252]

For all these reasons, with a vision of future studies for IBD therapies, it is important to consider the origin of the MSC.

### 5.2. Cell-Free Therapy

The therapeutic effect of MSC is mainly produced via paracrine signalling, soluble factors (growth factors and cytokines) and EVs. Therefore, MSC secretome may be considered a new therapeutic product [253] which can avoid some concerns derived from the use of stem cells themselves and offers several advantages: (i) easier to handle in the clinical practice; (ii) easier evaluation of safety, dose, and potency; (iii) storage without toxic cryopreservative agents; and (iv) its use is cheaper since it could be mass produced, stored and been available for a ready-to-use application [44,254].

The cell-free therapy modalities used in experimental models of IBD, include cell extracts, conditioned medium (CM) and extracellular vesicles (EVs).

#### 5.2.1. Cell Extracts

In DSS colitis mouse model, the intraperitoneally administration of UC-MSC cell extracts is more effective than UC-MSC to decrease activity index and histological scores, and to increase the body weight [72].

#### 5.2.2. Conditioned Medium

The secretome is composed by factors/molecules (soluble proteins, lipids or nucleic acids) and vesicles secreted to the extracellular space by the cells, which is obtained culturing MSC, this is why it is also named conditioned medium (CM). MSC-CM can significantly improve criteria of pathophysiology in different animal models and is usually as effective as MSC transplantation (Revised by [44]). MSC-CM administered intraperitoneally has demonstrated to improve the symptoms of experimental colitis and decrease TNF-α and MMP2 expression in mice [83].

#### 5.2.3. Extracellular Vesicles

EVs are membrane-bound phospholipid particles secreted by cells, containing RNA, proteins, chemokines, cytokines, integrins, and signal transduction factors, among others [47,255].

EVs can be classified as: (i) exosomes (30–120 nm); (ii) microparticles (150–1000 nm); and (iii) apoptotic bodies (500–2000 nm). EVs is a relevant intercellular communication pathway, acting through different mechanisms such as bind directly with a surface receptors to activate signalling pathway, or internalization trough membrane fusion or endocytosis process to release its content into the cytosol of the recipient cell [255].

EVs from MSC secretome are producing a great interest as a promising alternative to exploit MSC properties. Indeed, EVs show important advantages such as a less immunogenicity than MSC [256], the possibility of a high scale production and easier storage than MSC and a longer half-life in the bloodstream [257] and tropism towards inflamed tissues and tumors [258,259]. EVs have a similar immunoregulatory potential than MSC, but due to their small size, EVs are able to pass-through capillaries, enter the peripheral circulation and even cross the blood-brain barrier.

There are data indicating that EVs modulate macrophage polarization from M1-type to M2-type in the colonic tissue from DSS colitis mice through the enhancement of enhanced Arg-1 and CD206 expression in colon macrophages [260,261,262,263].

It has been also reported that MSC-secreted exosomes, by intraperitoneal injection, alleviates experimental-induced colitis (both DSS or 2,4,6-trinitrobenzenesulfonic acid (TNBS)-induced IBD mouse models) and reduced mortality in mice [100,102,136,264,265,266]. In these studies, EVs used were derived from BM, UC or AT tissues, and their administration improved the symptoms of UC by reducing weight loss, disease activity index, and colon mucosa damage and severity while increasing colon length. These therapeutic effects may be due, in part, to the anti-inflammatory and regenerative mechanisms induced by MSC-derived EVs.

On the other hand, it was revealed that the levels of VEGF-A, IFN-γ, TNF-α, IL-12, CCL-24, and CCL-17 and IL-17 were decreased, whereas the level of TGF-β, IL-4, and IL-10 were increased in lymph node and spleen of mice treated with exosomes. In addition, the percentages of CD4^+^ CD25^+^ Foxp3^+^ Treg cells were grown in these organ places [100]. Also, EVs-MSC induce the conversion of T helper type 1(Th1) into Th2 and decrease Th17 [266,267,268]. Also, EVs induce macrophage polarization to an M2 phenotype and promote UC repair through the JAK1/STAT1/STAT6 signalling pathway.

There are also data showing a protective effect of MSC-exosomes on the intestinal mucosal. After DSS or TNBS administration, the number of goblet cells in the colonic mucosa is significantly reduced, and it was shown that MSC-Exo injection significantly rescues the goblet cell population [266]. In addition, it was reported that intraperitoneal injection of MSC-Exo increased the expression of ZO-1, occludin, and claudin-1 in colon tissue and alleviates the disruption of the intestinal barrier.

Interestingly, it has also been reported that EVs-MSC restored the intestinal barrier function by improving mitochondrial dynamic balance [269]. This is also a relevant finding considering that mitochondria have a key role in energy metabolism, but are also involved in various pathophysiological processes, including cell apoptosis, and proliferation [270].

## 6. New Technologies for MSC Cultures and Mass Production of Secretome Derived Products

### 6.1. MSC In Vitro Production

MSC are widespread in many human organs and tissues, but in small numbers, so in vitro expansion is required for several weeks to achieve enough cells for cell-based therapies. Then, a large-scale expansion process is crucial to obtain enough cells for a therapy in a time and cost-effective manner. A large-scale production can be carry out using multi-layered flask, spinner flask, roller bottle, or bioreactor, which are used for expansion of MSC [271].

The use of bioreactors for MSC expansion allows to develop an industrial and commercial manufacturing and to obtain a monitoring of the expansion process, limiting batch-to-batch variability and fluctuations of cell culture conditions (pH, oxygen concentration, or nutrient gradients) caused by manual medium exchange.

Specific technological parameters must be considered to establish an expansion process in bioreactor such as the type of microcarriers, hydrodynamic parameters and agitation. Microcarriers are small beads (100 to 300 μm) made of diverse materials (polystyrene, dextran, cellulose, gelatine, glass, or decellularized tissue…) and used to increase the surface area available for cell attachment, providing a high surface-to-volume ratio for high-density cell culture with a cost reduction [272,273]. Recently, it was reported that, compared with 2D-cultured tonsil-derived MSC (TMSC), 3D-cultured TMSC showed a higher anti-inflammatory cytokine expression and induce a significant lower disease activity index score, body weight, colon length shortening and histological scoring index [274].

Compared with classical 2D cell culture systems [275], high scale-production is more advantageous, but there is the need to understand cell culture conditions which impact on MSC therapeutic effect [276].

### 6.2. Ex Vivo MSC Modifications toward More Specific Therapeutic Applications

Oxygen pressure, pH or cell pre-conditioning with inflammatory factors are ex vivo modifications that impact on MSC effect.

MSC are aerobic cells that need an adequate supply of oxygen; however, tissue oxygen saturation (5–7% O_2_) is at least 3 times lower than in standard cell culture flasks (21% O_2_) which can increase ROS production [277,]. Cell culture in hypoxic conditions can enhance MSC proliferation and decrease apoptosis [278], which suggest that it would be preferable to impose hypoxia during the expansion phase, to mimic the natural niche, instead of preconditioning MSC [279]. In addition, hypoxia can enhance pluripotent markers [280,281], cytokines and growth factors expression [282], improving MSC angiogenic capacity [283], anticancer effects [284] and their ability to migrate to the site of injury markers [285]. In this sense, it was reported that hypoxia-preconditioned MSC could reduce colon inflammation to a large extent compared with normoxia-preconditioned MSC in a DSS mouse model [286].

Usually, cell expansion is performed at 37 °C and neutral pH (7.2–7.4) it has been described [287] that pH can influence MSC metabolism which impact on their secretome and therapeutic effect. Studies about temperature and pH impact on MSC effect must be deeply evaluated.

MSC preconditioning with inflammatory cytokines, such as IFN-γ [288] and TNF-α [289] can improve therapeutic effect. Preconditioned MSC with IFN-γ and/or TNF-α, increase the expression of anti-inflammatory factors and therefore, improve the immunosuppressive function of MSC [290]. Also, preconditioning rat BM-MSC with licochalcone A (a flavoid compound extracted from legume liquorice) enhances MSC therapeutic activity in animal models of colitis, by increasing the number of MSC migrating to the inflammation site probably promoting CXCR4 expression [286].

It is also relevant the possibility to manipulate the EVs production by MSC in order to improve their anti-inflammatory properties. Thus, EVs from cultured medium of BM-MSC transfected with recombinant lentiviruses overexpress an anti-inflammatory miRNA (miR-146a), a well-known and which acts as a negative feedback regulator of the innate immune response. It was showed that these obtained EV significantly inhibited TNF receptor-associated factor 6 (TRAF6) and IL-1 receptor-associated kinase 1 (IRAK1) expression in TNBS-induced colitis of rats [291].

It has been also reported that EVs from thapsigargin (TSG-EV)-treated Warton’s jelly-derived MSC intraperitoneally injected ameliorated experimental colitis decreasing (or at least maintain similarly) pro-inflammatory factors expression (IFNγ, TNFα, and IL-1β) and inducing anti-inflammatory factors secretion such as TGFβ, COX-2 and IDO, up to 15 fold for this latter [292], which can induce the development of regulatory T cells and M2-type macrophages [293]. Indeed, TSG-EVs increase T cell proliferation and amount of Treg and M2-type macrophage and induce Th1 and Th17 differentiation. Thus, TSG-EV reduce the inflammatory response, maintain intestinal barrier integrity, and therefore, substantially alleviated colitis symptoms [292].

In addition, it was reported that UC-MSC cultured in a xeno-free medium showed an enhanced therapeutic effect in a mouse model for UC, compared with UC-MSC cultured in a conventional medium [294].

Another strategy is to engineer MSC to overexpressed specific factors to improve their therapeutic effect in IBD. In this sense, recently Zhou et al. showed that Nrf-2-modified hair follicle MSC improved DSS-induced UC in rats [11]. Nrf-2 is a transcription factor which regulates the expression of gene related to detoxification and anti-inflammatory response, among others [295].

The capacity of MSC and their secretome can be modulated through molecular or chemical stimuli, which suggest the interest to investigate these aspects to adapt the potentiality of MSC or their derivates to different therapeutic applications, such as IBD.

### 6.3. Standardization and Functional Tests Research for Specific Applications

Standardized criteria for the donor selection, transport, cell culture and storage of MSC during the manufacturing process are not established. Currently, allo-MSC from one or various donors are used as a universal drug for multiple patients; however, there is donor-to-donor, tissue source and cell culture strategies (with or without preconditioning) differences that can impact of clinical effectiveness [296,297,298,299]. Indeed, MSC expansion strategies impact deeply on their molecular phenotype compared to donor age [103,300,301,302], since the high-scale production of allo-MSC can minimize donor and bioprocessing variability, standardized practices should be established between laboratories/companies for MSC manufacturing and derived products, like the secretome. On the other hand, although MSC clinical effectiveness is in part due to the secretion of trophic factors, there is no established and standardized potency assays for the release of MSC- based therapy [303]. Thus, a functional characterization of allo-MSC or secretome is required for each therapeutic indication. In this sense, Caco-2 cells in vitro may be a biological platform to explore the functional capacity of the optimal MSC and their secretome products.

### 6.4. Route of Administration

The route of administration of MSC used in IBD were intravenous, intra-peritoneal or local. Intravenous administration was the most used in experimental studies in clinical trials. Despite the homing properties of MSC, a previous study has shown that less than 1% of MSC injected intravenously homing at the damaged intestinal tissue [304]. Another alternative route of administration in IBD is intraperitoneally, proven to be satisfactory in view of MSC migration capability towards the inflamed intestine and therapeutic effectiveness [305]. In addition, such as was referred more above, experimental studies demonstrated that EVs administrated by this way produced therapeutic effect in IBD [266,292]. Another possibility is the local administration which demonstrate positive beneficials in clinical trials for perianal fistulas in CD. In this sense, the follow-up study through week 104 of the clinical trial known as “Adipose derived mesenchymal stem cells for induction of remission in perianal fistulizing Crohn’s disease,” or ADMIRE-CD reported 7 treatment-emergent serious adverse events and clinical remission in 56% patients of the darvadstrocel group and 40% in the control group [306]. Regarding intraluminal lesions, the local administration through mucosa, that exhibit a high permeability but a low risk of overdose, seems to be a good option for drug delivery but the retention time is limited, and this is the main disadvantage and may be the cause of a less therapeutic effect [307,308]. Nevertheless, there is the possibility to combine the administration of MSC or their secretome-derived products with new technologies, such as mucoadhesive hydrogels. In this line, a recent study assessed the effect of rectal administration of a thermosensitive hydrogel loaded with CM from human uterine cervical mesenchymal stem cells (hUCESC-CM) in DSS-induced colitis mouse model [122]. Treatment hydrogel loaded with hUCESC-CM (H-hUCESC-CM) reduced body weight loss, gene expression of pro-inflammatory factors (TNF-α, IFN-γ, and IL-6) and colonic tissue damage such as atrophy, inflammation, and dysplasia. It has been reported that hydrogel alone can reduce pro-inflammatory cytokines expression, such as TNF-α and IFN-γ; probably due to polymers properties [309,310].

This last study shows the importance of the tissue source of MSC, the use of their secretome and the administration route using new technologies such as hydrogels. Human uterine cervical stem cells (hUCESC) are a characterized new population of MSC [180] isolated from the transformation zone of the human uterine cervix of healthy women [248] and whose secretome shows a potent regenerative, anti-inflammatory, anti-tumor and anti-microbial capacities [166,180,311]. On the other hand, hydrogels are employed due to their biocompatibility, similarity to the extracellular matrix, permeability for oxygen, small molecules and nutrients [312,313], converting them into optimal carriers of MSC secretome to restore tissue damages in IBD. Hydrogels can spread over colonic mucosa and release bioactive molecules from the MSC secretome, allowing a faster regeneration.

In this previous work [314], the hydrogel was developed using Pluronic^®^ and one hydroxypropylcellulose (MK4M). Pluronics^®^ are amphiphilic, thermosensitive, and bioadhesive and are mainly used to induce gelation (from liquid to solid state) [314,315]. MK4M is a viscous cellulose derivative used to generate mucoadhesion or even regulating the release of molecules. Therefore, the combination of both compounds improves hydrogel adhesion and protection to the colonic mucosa [316].

## 7. Conclusions

The complexity of IBD pathophysiology makes difficult to find a therapeutic strategy that effectively and globally addresses the different mechanisms implicated and clinical manifestations.

There is an evolution of the concept of the therapeutic interest of MSC: from the use of autologous to allogeneic MSC, from the first conception of their regenerative therapeutic effect to anti-inflammatory, and more recently to others such as anti-tumor and anti-inflammatory, anti-microbial or anti-oxidative stress. Likewise, there is an evolution from cell-based therapies to cell-free therapies based on their secretome-derived products. The development of all these evolutions could lead to the birth of a new therapeutic strategy for IBD.

However, for this, new challenges must be faced, such as conveniently integrating new approaches and technologies, such as the appropriate choice of the ideal source of MSC to counteract the different multifactorial pathophysiological mechanisms involved in IBD. In addition, for the implementation of the new products in the clinical practice, we must develop an adequate mass production and standardization process and the integration of new technological developments such as hydrogels that, based on nanotechnology, can optimize the therapeutic topic application. All this will allow us to design new therapeutic horizons for IBD based on biological therapy against its diverse and complex therapeutic targets.
